# Comparative Study between Physics-Informed CNN and PCA in Induction Motor Broken Bars MCSA Detection

**DOI:** 10.3390/s22239494

**Published:** 2022-12-05

**Authors:** Abderrahim Boushaba, Sebastien Cauet, Afzal Chamroo, Erik Etien, Laurent Rambault

**Affiliations:** University of Poitiers, ISAE-ENSMA Poitiers, 2 rue Pierre Brousse, TSA41105, CEDEX 9, 86073 Poitiers, France

**Keywords:** MCSA, PCA, deep learning, physically informed, PINNS, broken bars, fault detection

## Abstract

In this article, two methods for broken bar detection in induction motors are considered and tested using data collected from the LIAS laboratory at the University of Poitiers. The first approach is Motor Current Signature Analysis (MCSA) with Convolutional Neural Networks (CNN), in which measurements have to be processed in the frequency domain before training the CNN to ensure that the resulting model is physically informed. A double input CNN has been introduced to perform a 100% detection regardless of the speed and load torque value. A second approach is the Principal Components Analysis (PCA), in which the processing is undertaken in the time domain. The PCA is applied on the induction motor currents to eventually calculate the *Q* statistic that serves as a threshold for detecting anomalies/faults. Even if obtained results show that both approaches work very well, there are major differences that need to be pointed out, and this is the aim of the current paper.

## 1. Introduction

Induction Motors (IM) are commonly used nowadays. Aside from a wide use in modern industries, the application of IMs can be found in various sectors (military, medical, nuclear, etc.) [1,2]. These sectors require IMs to mostly operate in harsh conditions following rough cycles and long working hours, and these uneasy situations increase the probability of breakdowns and malfunctions. At the same time, precision and fidelity are essential for these sectors in order to guarantee optimal performance. As a matter of fact, IMs require regular monitoring and maintenance [3,4], a process also known as condition monitoring. In some cases, intrusive analysis is required. This type of analysis is often a waste of operating time for industries. In other cases, the diagnosis of faults depends only on the analysis of some recorded measurements, such as currents and vibrations of the IM [5], but this approach requires a high level of experience, which a machine operator usually does not have. The faults that occur in IMs are classified into mechanical, rotor based and stator based. The present paper deals with the rotor faults and, more precisely, the broken rotor bar (BRB) faults.

Different methods have been developed to detect broken rotor bar (BRB) faults without intrusion. These methods can be divided into two categories: the first category is based on statistics, while the second one is based on physical information. Statistical methods use time domain measures to characterise the healthy state of the motor with a set of statistical indexers or coefficients. This helps in identifying any abnormality in future measurements that indicate a fault. Some approaches tend to calculate a diverse set of coefficients from time domain measurements directly. The authors of [6] identify three statistical coefficients: Measure of Central Tendency (MCT), Measure of Variability (MV), and Measure of Dispersion (MD). In cases with large data sets, the Principal Components Analysis (PCA) method is used to reduce the dimensions of the data and focus only on the important ones by analysing the *Q* statistic (squared prediction error (SPE) in other sources) or the T2 Statistics [7,8]. The other set of approaches is based on selecting certain fault frequency characteristics, which are determined thanks to the knowledge we have on IMs [9]. The study in [9] offers a classification of different methods in order of significance for each fault type. From this review, we can see that Motor Current Signature Analysis (MCSA) is the most suitable one for our goal. The performance of the MCSA has been reported in the literature [1,10,11,12]. This approach aims to transform the measured signals into the spectral domain in order to select the characteristic frequencies generated by the fault’s periodicity. In some cases, the analysis needs more tools and techniques, such as the Hilbert transform [1], kurtosis selection of sub-bands [13], autogram selection of sub-bands [14], other than a simple Fourier transform. In recent works [15], the approach is based on the analysis of the startup transient current signal through the current signal homogeneity and the fourth central moment (kurtosis) analysis.

The MCSA approach has already been developed and put into use in different industries, but it still requires a certain level of knowledge and expertise to be used by machine operators [10,11,16]. The real challenge is to automate the analysis while ensuring the reliability and fidelity of the models. Different studies propose the use of Machine Learning (ML) and Deep Learning (DL) [17]. Algorithms such as Support Vector Machines (SVM), K-Nearest Neighbor (K-NN), and Artificial Neural Network (ANN) have proved to be very useful, especially with the huge memory and calculation performance of modern computers [18,19]. Data scientists and Artificial Intelligence (AI) specialists usually fit raw data collected from the IMs into their ML or DL models in order to obtain a diagnosing model for certain faults allowing fault severity supervision; the method in [18,20] shows high performance. Nevertheless, the aim of the present article is to introduce the physical knowledge we have on IMs in the process of training an Artificial Intelligence (AI) model [20]. The performance of these tools is based on their ability to extract useful features and to detect patterns. The previously mentioned analysis method (MCSA) is used to only select the relevant fault information prior to the training process. Furthermore, the *Q* statistic method based on PCA also showed a high performance in the detection of rotor faults, as mentioned in [7]. The advantage of this approach is that the detection of faults and their severity can be assessed to the comparison of the Q mean of a signal with previously identified thresholds calculated from healthy IMs, making it easier for operators to diagnose IMs without the need for sophisticated algorithms such as DL and ML. A first approach in [21] uses the Neural Network, but this method is not as efficient as recent Convolutional Neural Networks-based methods.

We can see that Neural Networks (NN) are widely used to solve this problem [9,18,22]. In this article, a physical-informed Convolutional Neural Network is used, and a new double input CNN is introduced to obtain a better fault detection efficiency. The advantage lies in the convolutional layers that treat the inputs or the physical signals with different filters in order to diversify the features, which are then used to train the dense layers. This approach helps the model to achieve good generalisation while preventing over-fitting [9].

CNN and PCA approaches have shown great results in former articles, but in this study, we are going to use the same dataset to explore their performance and compare them in terms of reliability, execution time, precision and embedding difficulty. This comparison can be used to choose the right approach for industrial applications. Engineers will be able to identify the needed performance for their ongoing application and pick the suitable method.

## 2. Broken Rotor Bar (BRB) Fault

**Figure 1:**
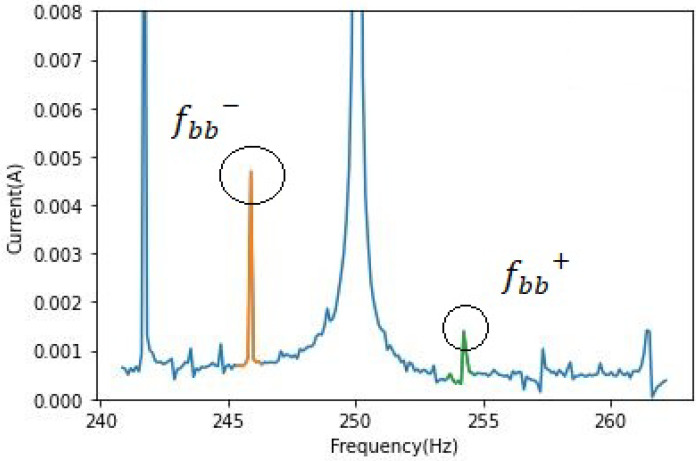
Phase current IM with broken bar fault, zoom on the fifth harmonic.

Broken Rotor Bar (BRB) faults account for 5% to 10% of total IMs faults. The main reason is the high torque demand and thermal stress. When a bar is broken, the internal flow of current will be obstructed. As a result, there will be a lack of field near the faulty bar in the rotor. Due to this imbalance, an Unbalanced Magnetic Pull (UMP) is created, which rotates at the speed of the motor. It modulates at a frequency that is equal to a multiple of the slip frequency. This frequency, known as pole pass frequency, causes increased magnitude in the vibration spectrum and occurs at the rotational frequency [19]. The BRB faults are more important than other faults because of the massive damage and consequences. In the case of broken bars, the generated components are modulated and carried by the supply frequency $f_{s}=$ 50 Hz (in Europe) and its odd harmonics. The broken bars’ characteristic frequencies $f_{bb}$ can be identified by the following equation [23]:

(1) $$f_{bb}=\left(v\pm 2ks\right)f_{s}$$

where *s* is the slip, *v* = 1, 2, 3, … and *k* = 1, 2, 3, …. As mentioned in [24], it can be quite hard to identify faulty rotors in the case of a light load because the slip is rather small, and the fault frequency is not only close to the fundamental component but is of a relatively small amplitude. In each of the following figures, as viewed in Figure 1, the ${f_{bb}}^{-}$, in orange, and ${f_{bb}}^{+}$, in green, represent both fault frequencies around fundamental and harmonic frequencies.

**Figure 2:**
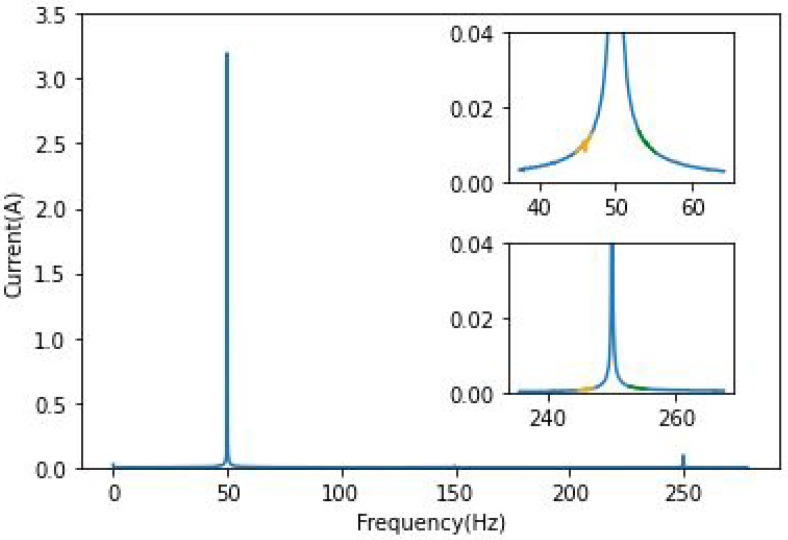
FFT phase current of healthy IM/Zoom around 50 and 250 Hz.

The characteristic frequencies on the left side of the harmonics or the fundamental frequency are displayed in orange, and the ones on the right are displayed in green to make the amplitude difference clearer. Figure 2 corresponds to the healthy cases around both frequencies of the phase *a*.

**Figure 3:**
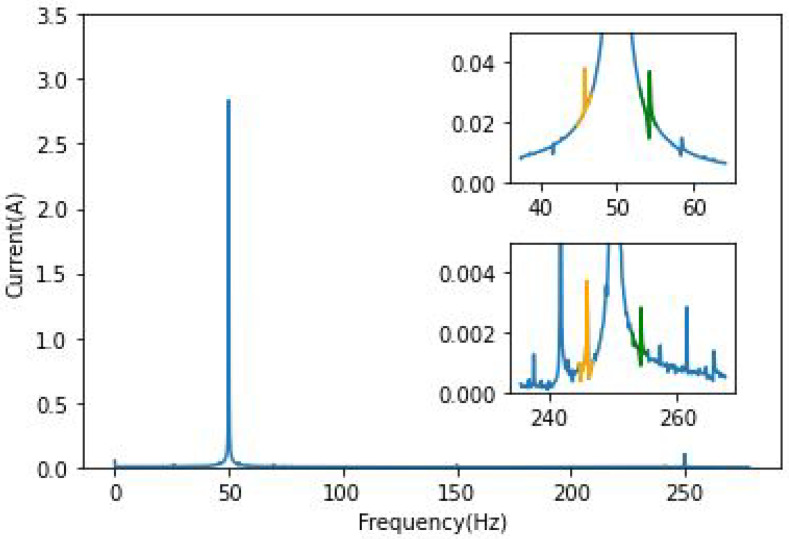
FFT phase current of 1BRB IM/Zoom around 50 and 250 Hz.

**Figure 4:**
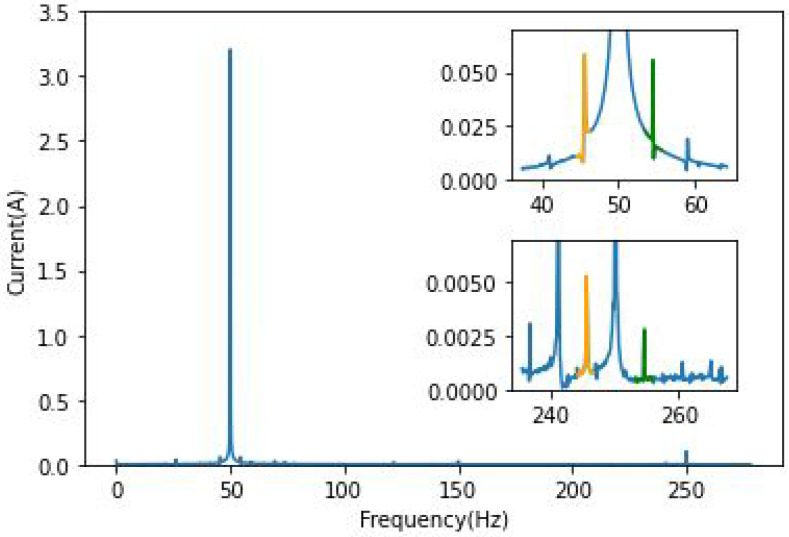
FFT phase current of 2 BRB IM/Zoom around 50 and 250 Hz.

In order to illustrate the results, the frequencies around the fundamental 50 Hz and the fifth harmonic 250 Hz have been chosen. In this case, this harmonic is the easiest component to be followed for BRB. Figure 3 and Figure 4 clearly show the effect of broken bars on the spectrum of phase “*a*” current; a primary analysis of its frequency domain has shown that the fault is visible only around the fundamental component and the fifth harmonic. As presented in these figures, the amplitude of the characteristic frequencies is proportional to the severity of the damage. This way of highlighting the fault components using the Fourier transform is the most basic amongst MCSA tools. The squared envelope (which can be described as a benchmark method for faults analysis) can be used to extract fault-relevant features.

In Figure 2, we can see that there are no fault components since the rotor is balanced.

Single and double broken bar fault components are visible in Figure 3 and Figure 4 around the 250 Hz frequency, with an increase in amplitude whenever the load is increased. It can be noted that the left and right components are not of the same amplitude.

## 3. First Approach: Physics-Informed CNN Based on MCSA

The Motor Current Signature Analysis (MCSA) is considered one of the most efficient approaches in IM’s fault diagnosis. Typically, Fourier transform is applied to the IM current signals, thus allowing diagnosis through fault components analysis. Sometimes more treatment has to be applied due to noise and modulation, usually resulting in calculating the squared envelope of the signal using the Hilbert transform. Henceforth, the fault components would be clear in the resulting signal’s spectrum.

### 3.1. Spectrum Analysis

The application of a Fourier transform on one of the currents will give a representation in the spectral domain. The most dominant frequencies correspond to the supply frequency 50 Hz and its harmonics (i.e., 250 Hz), as shown in Figure 1. As said previously, the fault components are modulated, and their carriers are the supply frequency and its odd harmonics. By analysing the spectrum of different samples that each represent a different severity, we observed that the components on the left of the carrier are not equal in amplitude to the ones on the right. It would also be interesting to take into consideration the whole sub band of frequencies corresponding to 5% of $f_{bb}$ to ensure picking all fault information that might move due to inaccuracy in calculating the slip: 5% was chosen because it gave the best performance in the previous studies [20]. It can be observed that the amplitude of the component increases proportionally with the severity of the damage and that the component does not exist in the healthy state. The second observation should be enough for a diagnosis, and the first one can be used to add more diversity to the training data for the CNN that we will discuss after.

The input of the CNN should be the sub-bands containing fault components. In other words, from each spectrum, we take the 5% sub-bands around the $f_{bb}$ with *v* equal to 1 and 5 and feed them as a sample to the CNN model.

### 3.2. Squared Envelope Analysis

**Figure 5:**
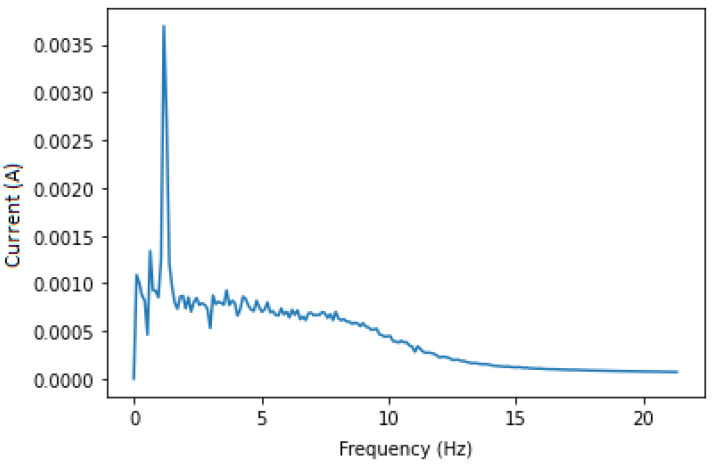
Current envelope of faulty IM, 250 Hz.

**Figure 6:**
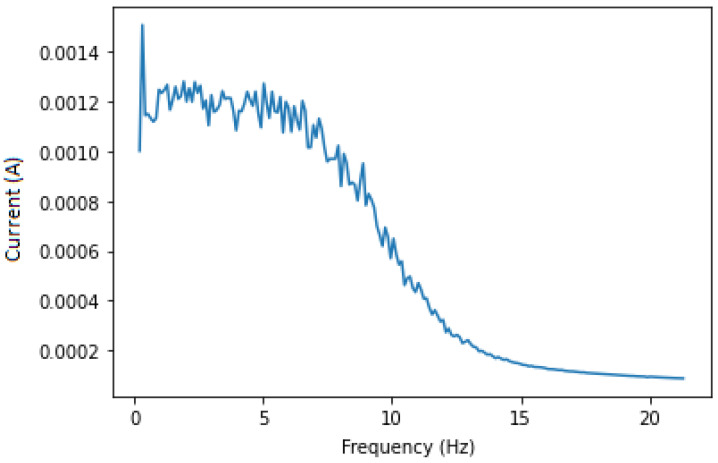
Current envelope of healthy IM, 250 Hz.

**Figure 7:**
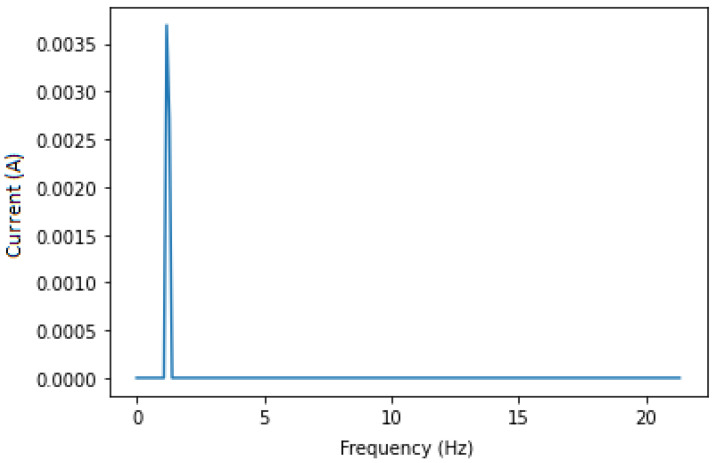
Squared envelope of faulty IM after threshold.

This approach aims to isolate the fault components for the CNN (or the expert analysing the IM) to obtain a clearer set of data, which is the sub band of frequencies from 0 to 20 Hz that always contains fault components no matter the variation in speed, load or IM dimensions. Applying a band pass filter around 230 and 270 Hz is essential to remove most of the unwanted noises, thus isolating the needed components only. In this case, the 250 Hz harmonic and fault components around it are chosen to avoid noise and any other type of frequencies that are near 50 Hz. After filtering, we apply the Hilbert transform in order to calculate its squared value to end up with a signal containing only the fault components at $f_{bb}=\left(2ks\right)f_{s}$. The squared envelope is shown in Figure 5 and Figure 6 for both healthy and faulty IM. Even if there is some noise, the figures show expected results. Knowing that a healthy IM does not have $f_{bb}$ components, we analysed the whole data set in order to find the maximum amplitude in the squared envelope of healthy IMs, which is 0.0015. A threshold with this value has been applied to eliminate remaining noises, and the result featured in Figure 7 is satisfying.

### 3.3. Description of Dataset

**Figure 8:**
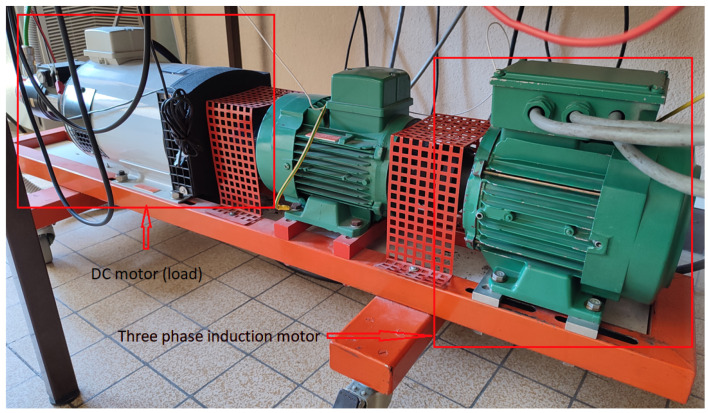
LIAS experimental bench, green IM to the right is the induction motor and the grey one is the DC motor, green motor in the middle is not used.

The dataset used in this study was generated in the LIAS laboratory of Poitiers. The experimental bench in Figure 8 was set up by Pr. Gérard Champenois from the University of Poitiers. It consists of a three phases induction motor (see Table 1), a DC motor that acts as a load, an Altivar 58f drive and a dSPACE control board. Table 2 shows the experiment files containing: currents, voltages and time measurements. Different recordings were established corresponding to different loads with speeds close to the nominal functioning of the induction motor since the PLC was not used for control—the IM was supplied directly with necessary power.

**Figure 9:**
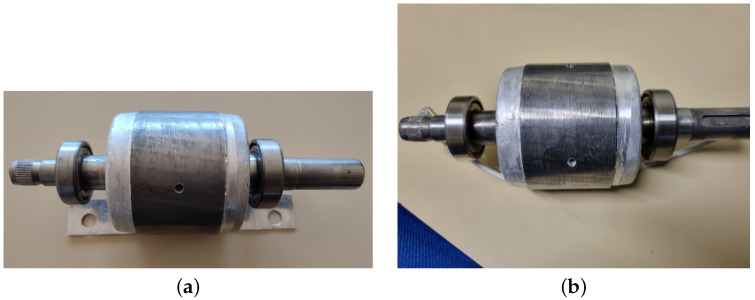
(**a**) On the left, 1 BRB: rotor with one hole. (**b**) On the right, 2 BRB: rotor with two holes.

The dataset is split into three health classes: “Healthy”, “one broken rotor bar (1BRB)” and “two broken rotor bars (2 BRB)”, as shown in Table 3. A broken bar is considered as a hole in the rotor (Figure 9a). For the two broken bars class, two holes were drilled in 0 and 6 o’clock positions (Figure 9b). As shown in Table 4, for each class, three different loads were applied, resulting in three speed ranges that differ from class to class, 1432–1439 rpm for high load, 1458–1468 rpm for medium load and 1490–1492 rpm for low load.

**Figure 10:**
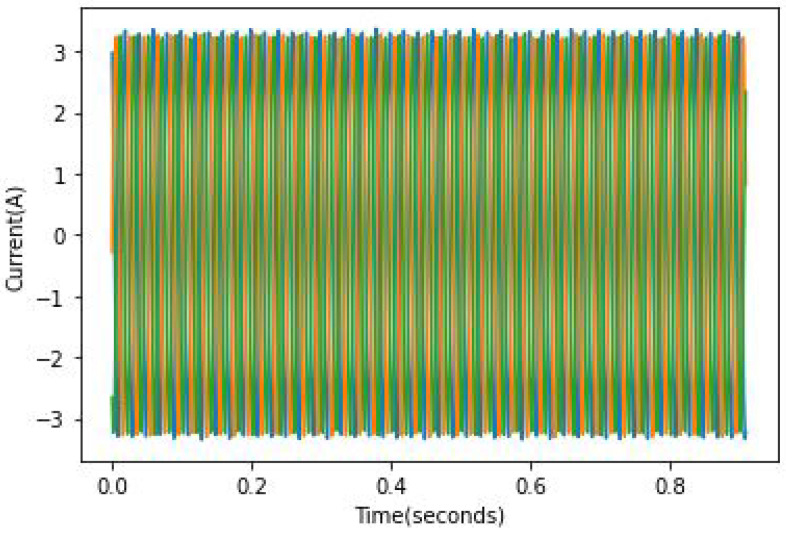
$i_{a}$ current in a healthy case at full load.

**Figure 11:**
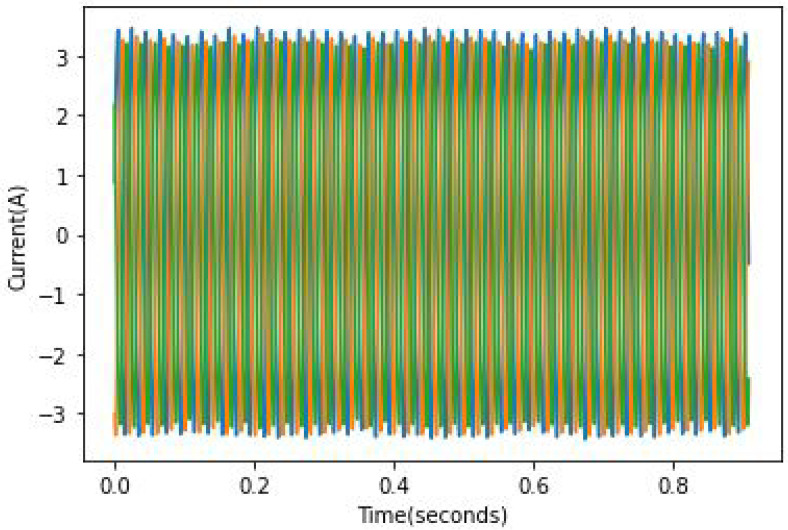
$i_{a}$ current in 1 BRB case.

**Figure 12:**
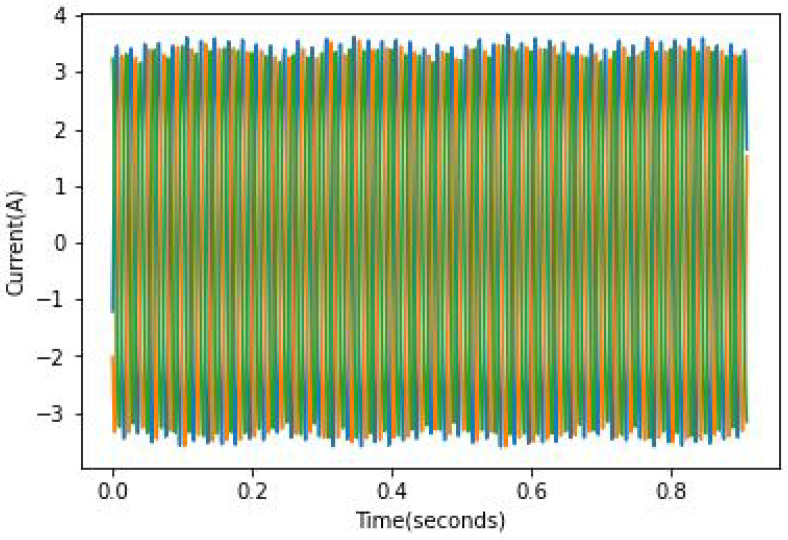
$i_{a}$ current in 2 BRB case.

The speed and load were constant throughout all experiments. A variation in speed and load from one experiment to another allows the testing of the model’s robustness because the fault effect is proportional to the load, which means the lower the load, the harder it is to spot the fault. As mentioned in [24], it can be quite hard to identify faulty rotors in the case of a light load because the slip is rather small and the fault frequency is not only close to the fundamental component but is of relatively small amplitude. An example of full load current records can be found in Figure 10, Figure 11 and Figure 12. In these figures, the broken bar influence can be seen on the current envelope.

The motor was connected to a 50 Hz supply, and the connection between the bench and the Matlab software was established using the dSPACE card.

Half of the recordings were designated for the model training, and the other half was used for testing.

Tests are divided into three categories corresponding to the three speeds:

- Test 1 is low speed (highest load);
- Test 2 is medium speed (medium load);
- Test 3 is high speed (lowest load).

This will allow us to validate the model reliability and robustness in different working conditions.

### 3.4. CNN Model

#### 3.4.1. Convolution Neural Networks (CNN)

**Figure 13:**
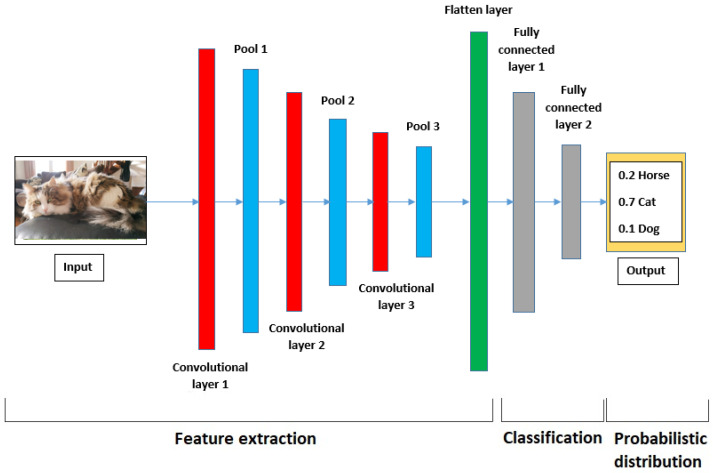
General CNN structure for animal classification (horse, cat and dog).

CNNs have found their application in different sectors such as bio-medical and image recognition because they were originally built to mimic biological neuron response [25], but their application for fault detection is quite recent. CNNs are a type of feed-forward artificial network, which has the advantage of automatic feature extraction thanks to convolution layers. Several structures have been developed for image recognition, such as LeNet-5, VGG16, and ResNet, but their application with IM signals has not proven to be successful. There are different types of layers besides the convolution ones, the pooling layer (PL), batch-normalisation layers (BN) and the fully connected layers (FCL) [18]. Figure 13 illustrates a CNN example for animal classification.

Furthermore, the convolution layers are usually defined with kernels (number and size), which are used to parse data coming from the input layer using a nonlinear activation, which is usually the rectified linear unit (ReLU) function. Further details can be found in [9]. The pooling layers come after the convolution layer (CL), the primary objective is to reduce the size of CLs output as well as to prevent over-fitting. Batch-normalisation layers are used to scale the batches for more model robustness and to speed up the training process. The last set of layers in a CNN contains the FCLs, which are typical feed-forward layers that use the result of the convolution and pooling to train the structure and find the neural network (NN) weights.

#### 3.4.2. One-Dimensional CNNs

CNNs were originally built for 2D inputs, but they can be used for 1D signals as well. The main difference is in the sliding of filters. For 1D CNNs, the sliding is done in one direction with respect to strides, but for 2D CNNs, the filter slides both vertically and horizontally. One-dimensional data can be easily turned into 2D in order to apply the usual CNN procedure. However, 1-D CNNs have many advantages:

- Low computational time and complexity.
- Smaller number of parameters allowing easier training of the NN.
- The direct application due to the compatibility of 1D with time, so there is no need to adapt the time-based signals.

#### 3.4.3. Pre-Processing of Input

Data processing and rehabilitation is an important task that directly impacts the performance of a deep learning model. Some of the extracted features would be irrelevant to the fault. A proposed solution is to pre-process the signals in order to extract the fault characteristic frequencies as mentioned in Section 3.1 and Section 3.2. Samples used to train the CNN are considered as an array that contains only fault-relevant information. By feeding this input structure into the CNN, the feature extraction focuses only on fault-relevant information, other than noise and normal functioning frequencies that can lead the learning process of the model to confuse the classification and produce errors that are not acceptable in the industrial domain. This model is usually referred to as CNNS.

**Figure 14:**
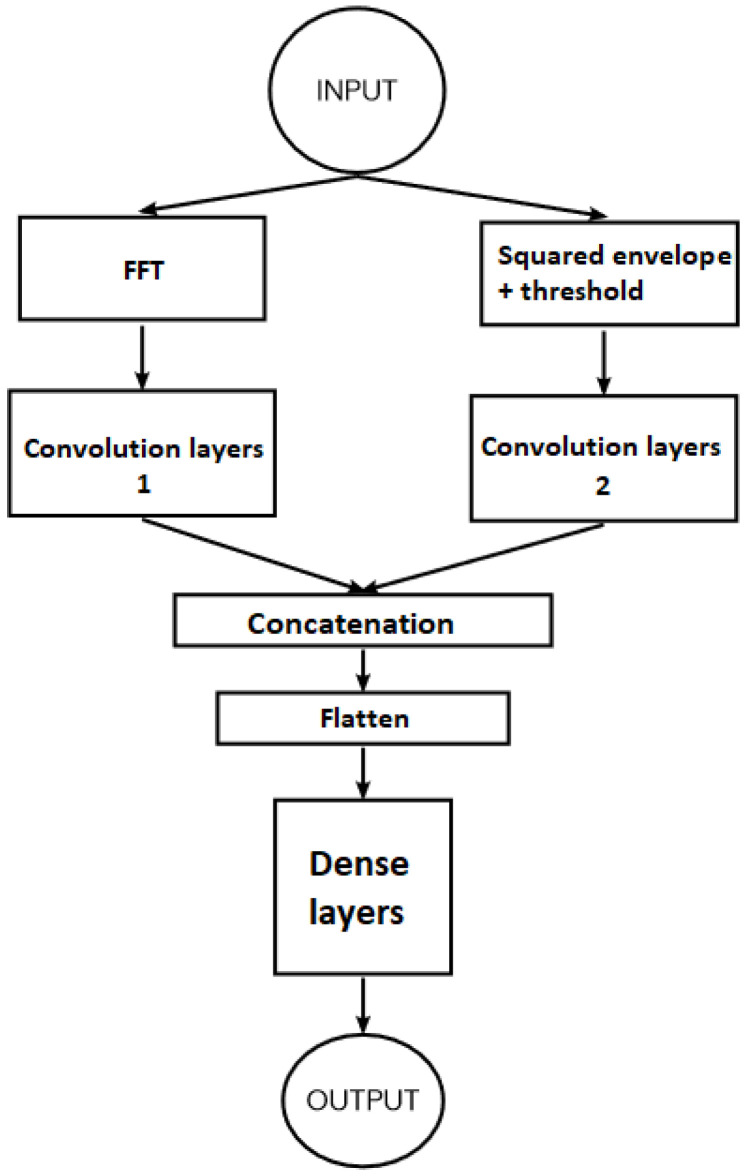
Structure of the DICNN.

The first attempt was to apply a CNN-1 using a single input processed with Fourier transform as presented in Section 3.1, and a CNN-2 using a single input processed with Squared envelope analysis as presented in Section 3.2. CNN-1 and CNN-2 configurations are given in Table 5 and Table 6. Confusion matrices are shown in Table 7 for CNN-1, Table 8 for CNN-2 and Table 9 for DCNN. Comparative results are displayed in Table 10, which shows that the CNN-1 has a perfect score in tests 1 and 3 only, and CNN-2 has a perfect score in test 2 only. In light of these observations, we can see that the union between CNN-1 and CNN-2 results will give a 100% score in all tests. The innovation in this paper is in the double input CNN model (DICNN), which uses both CNN-1 and CNN-2. A hypothesis was made that both input vectors contain different sets of information, which will help the DICNN obtain a diverse set of features of better quality. This is similar to the data augmentation technique used in image classification whereby data size and features are increased by analysing the existing input with different methods (usually rotating the pictures, as well as transforming them to black and white, etc.). Figure 14 represents the structure of the DICNN.

Each input has its own set of convolutional layers corresponding to the structure and configuration of CNN-1 and CNN-2. The output of these convolution layers is then concatenated to form one set of features. After that, the resulting matrix is flattened in order to be fed to dense layers for training. Dense layer configuration is presented in Table 11.

### 3.5. Results and Discussion

The results of DICNN are quite impressive. It can be seen that the model was able to generalize the problem perfectly due to the variety of features.

By analysing the results of these three models, we can make the following observations. First, the data processing using only Fourier transform gives good accuracy in the distinction between the severity levels of the damage, but there is the problem of confusing a healthy IM with faulty ones in some samples. This mistake is not acceptable for industries because it obstructs the production with false alarms. The second observation comes to the processing with squared envelope analysis; this approach offered a perfect distinction between healthy and faulty samples, but it has low accuracy in diagnosing the severity of the fault. Finally, the DICNN was able to gather both advantages of previous models to end up with a perfect fault classification and damage severity assessment.

Further investigation of this model’s performance should be undertaken using more datasets to ensure that this approach has a consistent performance.

### 3.6. Computational Efficiency

The hardware used for these tests is the default Google Colab hardware, which offers: 2vCPU @ 2.2GHz, 13GB RAM, 100GB Free Space. Time taken by the different task in each model are shown in Table 12.

The duration of each needed sample is 9.3429 s with a sampling time of 0.0007 s. The duration of the samples is long due to the small sampling frequency, but still, it allows for good online observation. The CNN-1 model has the fastest prediction time, and the DICNN is the slowest because it has more parameters and a more complex structure. The presented performance can be suitable for industrial use knowing that a broken bar fault is not fatal when it first appears, which makes the total prediction time acceptable.

## 4. Second Approach: PCA and Q Statistics

The fault detection process with statistic tools is the simplest amongst all the approaches proposed in the literature. If a fault occurs, it will disturb the normal functioning of an IM. This results in changes in the measured signals, thus allowing the use of approaches known as ‘novelty’ that detect the deviation of signals from their healthy state. Different methods can be applied to the fault detection problem: boundary methods, density methods, and reconstruction methods, etc. [7]. In this work, we chose one of the simplest reconstruction methods, which is the Principal Components Analysis (PCA).

The idea of PCA is to project *n*-dimensional data onto *k*-dimensional (n>k) hyperplane, thus minimising the projection distance from each sample point to the hyperplane and maximising the variance. As described in [26], the implementation of PCA mainly includes five steps:

- Standardise the data samples by mean normalisation.
- Calculate the covariance matrix of the data.
- Find the eigenvalues and the eigenvectors of the covariance matrix above.
- Combine the obtained eigenvectors according to the size of the eigenvalues to form a mapping matrix, and extract the largest number of the top *k* rows or the top *k* columns of the mapping matrix as the final mapping matrix.
- Map the original data with the mapping matrix of the previous step to achieve the purpose of data dimensionality reduction.

The anomaly detection procedure is based on the reduction in data dimensions. The *N* resulting PCA components are divided into two groups:

- The first group is made of the *M* components that describe the systematic trend of the system.
- The second group is called the residual space, which captures the noise trends.

Since faults are considered small changes in the measurement data, they are more likely to be found in the residual space. To detect this deviation, the *Q* statistic is applied for monitoring. The *Q* statistic is an indication of the distance of the specific data point from the principal component plane [27]. It can be easily calculated using the following formula:

(2) $$Q\;=\;r^{T}r\;\;\;\;\;\mathrm{where}\;\;\;\;\;r=\left(I-VV^{T}\right)x$$

where *x* is one of the IM currents and *r* is the residual vector, which can be derived by projecting the input vector *x* onto the residual space. Furthermore, *V* is a d×M matrix (*d* is the current segment length) whose columns correspond to the principal components associated with the *M* largest eigenvalues.

The algorithm that is going to be applied is as follows:

- Step 1: the healthy data samples are divided into segments of equal width.
- Step 2: the PCA is applied to each segment.
- Step 3: the *Q* statistic is calculated for each segment.
- Step 4: the $Q_{mean}$ is calculated using the values from the previous step.

The detection is established by applying the same steps 1, 2 and 3, thus enabling comparison of the resulting *Q* statistic with the $Q_{mean}$. If it is larger, that means the motor is faulty; otherwise, it is healthy.

### Results and Computational Efficiency

In the case of IMs, the inputs are the three currents $I_{a}$, $I_{b}$ and $I_{c}$ giving us N=3. The *Q* obtained using Equation (2) from healthy data can be used to calculate $Q_{mean}$, which serves as a threshold for fault detection. The learning or threshold definition process is achieved by calculating the $Q_{mean}$ statistic, as mentioned before for a set of healthy IM data. Different segment widths were used to build the model: 150 points, 300 points, 400 points, 600 points and 800 points. The results are given in Table 13.

The table shows that the reliability of this method is optimal for a segment width of 600 points. Increasing the segment width or decreasing it shows that the success rate decreases; hence, the choice of 600 was made. The duration of the 600 points width segment is 0.42 s, and the prediction time for each sample is 0.027 s, which means that we can obtain a diagnosis every 0.447 s. The number of false alarms is quite small but still their existence is inconvenient for industrial applications, knowing that it might increase the users operation time.

## 5. Comparison

In the field of IMs fault detection, the most important criteria for industries are: precision, reliability, execution time and the difficulty of deployment in industrial environments. These criteria have a direct impact on the cost of service and maintenance. The precision of the DICNN is clearly better than that of the PCA’s, basing this conclusion on the dataset we have. Furthermore, the DICNN is more reliable due to its reliance on the physical knowledge and isolation of fault information, which the PCA approach lacks, making it more susceptible to mistakes in the presence of noise. Another advantage of the DICNN over PCA is the absence of false alarms when validating with our data. It is true that enlarging the data set or changing other parameters such as the motor type, size or fault position and severity can affect the model’s score, but the first obtained results show that even with these different changes, the DICNN remains superior. Moreover, throughout this study, we can conclude that the first approach can give an accurate prediction every 9.5329 s, while the second approach needs only 0.447 s. The DICNN has a relatively high execution time, but it remains acceptable in industrial operations. As for the application in industries, it is easier to embed the mathematical calculation of the PCA and *Q* statistic that needs time domain data gathered by a sensor rather than a whole CNN with different processing approaches. Finally, the feature that is offered by the DICNN only is the classification of fault severity. This is mostly helpful in the scheduling of maintenance since it offers more flexibility for operators.

## 6. Application of Motor Models

Motors used in industry today come with different characteristics and sizes. The idea of fault detection and monitoring will always be the same, either by finding differences in the measured signals or finding fault signatures that can indicate the presence and gravity of damage. The PCA method is somehow limited as it is applicable only to motors sharing the same characteristics. In order to obtain a monitoring model, the data have to be collected from a motor close in characteristics to the target. The damage can be either represented by holes in the rotor bars or by overworking the motor. The recording of data has to be under similar working conditions (speed and load). The CNN models discussed in this article are more adaptable to size change. This approach analyses the location of the fault directly, and upon finding the fault signature, the motor’s diagnosis can be easier. Using a scaling technique that normalises the model input signals from 0 to 1, for example (sometimes from −1 to 1), makes this approach more effective and adaptable to a larger band of motors with different sizes. This study based the analysis only on the fundamental frequency and the fifth harmonic. For more precision and to make sure that the model will detect faults in different locations and for a larger motor band, other harmonics from second to fourth can be added so as to avoid missing any other fault signature.

## 7. Conclusions

As mentioned in [24], it is hard to detect faults in the case of a low load because a slip is rather small. In this study, we have presented two approaches for the detection of broken bar faults under different loads. The results clearly show that DICNN is more precise and reliable, offering a severity assessment of the IM, which is not available with the PCA’s approach. On the other hand, the execution time needed for PCA is quite small compared to that of DICNN, and on top of that, its embedding in industries is easier given thee huge differentiation criteria that can determine which method is better for a specific application. However, the PCA approach has a major disadvantage when it comes to applying the resulting models to different motor sizes. DICNN covers a larger band application and the model can work with different motor sizes thanks to the normalisation, making the DICNN more suitable for general application, where PCA can only be used for specific applications. It is possible in some cases to not be able to ensure treatment in a steady state, in particular, when the load or the speed varies in time. In this case, as shown in [15], it is necessary to analyse a non-stationary signal. This represents perspectives on the work present in this article.

## Figures and Tables

**Table 1:** Induction motor characteristics.

Power	1.1 kW
Nominal speed	1435 rpm
Nominal current	2.6 A
Power factor	0.85
Nominal voltage	380 V

**Table 2:** Description of broken bars dataset.

Speed (RPM)/Fault	Healthy	1 BRB	2 BRB
low (1432–1439)	18 recordings	8 recordings	8 recordings
medium (1458–1468)	18 recordings	18 recordings	8 recordings
high (1490–1492)	18 recordings	18 recordings	18 recordings

**Table 3:** Broken rotor bar dataset.

Healthy	1 BRB	2 BRB
no hole	1 hole	2 holes at 0 and 6 o’clock

**Table 4:** Load data set.

Low Load	Medium Load	Full Load
less than 10% Full load	around 40% Full load	80% Full load

**Table 5:** CNN-1 configuration of convolution layers.

Layers	Filter Size	Filters Number	Strides Size
Input	1 × 96	-	-
conv1	1 × 4	20	1
conv2	1 × 10	16	1
conv3	1 ×16	12	1
conv4	1 × 20	8	1
conv5	1 × 32	4	1

**Table 6:** CNN-2 configuration of convolution layers.

Layers	Filter Size	Filters Number	Strides Size
Input	1 × 96	-	-
conv1	1 × 4	20	1
conv2	1 × 10	16	1
conv3	1 × 16	12	1
conv4	1 × 20	8	1
conv5	1 × 32	4	1

**Table 7:** Confusion matrix of the CNN-1 model with fault type classification.

CNN-1									
**Tests:**		**1**			**2**			**3**	
**Predict.\Actual.**	**H**	**1B**	**2B**	**H**	**1B**	**2B**	**H**	**1B**	**2B**
H	9	0	0	6	0	0	9	0	0
1B	0	9	0	3	9	0	0	9	0
2B	0	0	9	0	0	9	0	0	9

**Table 8:** Confusion matrix of the CNN-2 model with fault type classification.

CNN-2									
**Tests:**		**1**			**2**			**3**	
**Predict.\Actual.**	**H**	**1B**	**2B**	**H**	**1B**	**2B**	**H**	**1B**	**2B**
H	9	0	0	9	0	0	9	0	0
1B	0	9	3	0	9	0	0	9	3
2B	0	0	6	0	0	9	0	0	6

**Table 9:** Confusion matrix of the DICNN model with fault type classification.

DICNN									
**Tests:**		**1**			**2**			**3**	
**Predict.\Actual.**	**H**	**1B**	**2B**	**H**	**1B**	**2B**	**H**	**1B**	**2B**
H	9	0	0	9	0	0	9	0	0
1B	0	9	0	0	9	0	0	9	0
2B	0	0	9	0	0	9	0	0	9

**Table 10:** Accuracy results of the broken bar classification models. (See speed values in Section 3.3).

Inputs	Test 1: Low Speed	Test 2: Medium Speed	Test 3: High Speed	Mean
CNN-1	100%	88.88%	100%	96.29%
CNN-2	88.88%	100%	88.88%	92.58%
DICNN	100%	100%	100%	100%

**Table 11:** DICNN configuration of dense layers.

Layers	Filter Size	Filters Number	Strides Size
FCL	256	-	-
FCL	64	-	-
FCL	3	-	-

**Table 12:** Time taken by each model for different tasks.

Approach	CNN-1	CNN-2	DICNN
Training time (s)	13.5	6.0	14.8
Processing time per sample (s)	0.1818	0.1700	0.1840
Prediction time per sample (s)	0.0024	0.0039	0.0065
Total prediction time per sample (s)	0.1842	0.1739	0.1900

**Table 13:** Results of PCA model using different segment widths.

Segment width (points)	150	300	400	600	800
Duration (s)	0.11	0.21	0.28	0.42	0.56
Number of false alarms	1319/4752	33/2376	21/1782	9/1188	10/864
Percentage of success	72.24%	98.61%	98.82%	99.24%	98.84%

## Data Availability

https://www.lias-lab.fr/mcsa (accessed on 20 October 2022).

## Abbreviations

The following abbreviations are used in this manuscript:

**Table of variables**	
Variable	Description
$f_{bb}$	broken bar fault characteristic frequency
*s*	slip
*v*	order of supply frequency harmonic
*k*	order of fault component harmonic
*V*	matrix gathering the PCA with the M largest eigenvalues
*x*	input vector
*M*	systematic trend of the system
*d*	segment length
*r*	residual vector
*Q*	*Q* statistic
$Q_{mean}$	the mean of *Q* statistics calculated from the training data set
**Table of abbreviations**	
MCSA	Motor Current Signature Analysis
CNN	Convolutional Neural Networks
PCA	Principal Components Analysis
IM	Induction Motors
MCT	Measure of Central Tendency
MV	Measure of Variability
MD	Measure of Dispersion
SPE	squared prediction error
ML	Machine Learning
DL	Deep Learning
SVM	Support Vector Machines
K-NN	K-Nearest Neighbour
ANN	Artificial Neural Network
BRB	Broken Rotor Bar
UMP	Unbalanced Magnetic Pull
PL	Pooling Layer
BN	Batch Normalisation layers
FCL	Fully Connected Layers
CL	Convolution Layer
ReLU	rectified linear unit
NN	Neural Network
CNNS	CNN with processed input that contain only fault-relevant information
DICNN	Double Input CNN
CNN-1	CNN trained using data processed with Fourier transform
CNN-2	CNN trained using data processed with squared envelope analysis
